# The Insecticidal Activity of *Azadirachta indica* Leaf Extract: Optimization of the Microencapsulation Process by Complex Coacervation

**DOI:** 10.3390/plants12061318

**Published:** 2023-03-14

**Authors:** Mariela R. Michel, Mayra Aguilar-Zárate, Romeo Rojas, Guillermo Cristian G. Martínez-Ávila, Pedro Aguilar-Zárate

**Affiliations:** 1 Engineering Department, Instituto Tecnológico de Ciudad Valles, Tecnológico Nacional de México, Carretera al Ingenio Plan de Ayala Km. 2, Col. Vista Hermosa, Ciudad Valles 79010, San Luis Potosí, Mexico; 2 School of Chemistry-CIEP, Autonomous University of San Luis Potosí, Av. Dr. Manuel Nava 6, Zona Universitaria, San Luis 78210, San Luis Potosí, Mexico; 3 Chemistry and Biochemistry Laboratory, School of Agronomy, Autonomous University of Nuevo León, General Francisco Villa S/N, Ex-Hacienda “El Canadá”, General Escobedo 66050, Nuevo León, Mexico

**Keywords:** neem, *Tenebrio molitor*, Taguchi methodology, scanning electron microscopy

## Abstract

The objective of the present work was to optimize the microencapsulation conditions of neem (*Azadirachta indica* A. Juss) leaf extracts for the biocontrol of *Tenebrio molitor*. The complex coacervation method was used for the encapsulation of the extracts. The independent factors considered were the pH (3, 6, and 9), pectin (4, 6, and 8% *w*/*v*), and whey protein isolate (WPI) (0.50, 0.75, and 1.00% *w*/*v*). The Taguchi L9 (3^3^) orthogonal array was used as the experimental matrix. The response variable was the mortality of *T. molitor* after 48 h. The nine treatments were applied by immersion of the insects for 10 s. The statistical analysis revealed that the most influential factor on the microencapsulation was the pH (73% of influence), followed by the pectin and WPI (15% and 7% influence, respectively). The software predicted that the optimal microencapsulation conditions were pH 3, pectin 6% *w*/*v*, and WPI 1% *w*/*v*. The signal-to-noise (S/N) ratio was predicted as 21.57. The experimental validation of the optimal conditions allowed us to obtain an S/N ratio of 18.54, equivalent to a *T. molitor* mortality of 85 ± 10.49%. The microcapsules had a diameter ranging from 1–5 μm. The microencapsulation by complex coacervation of neem leaf extract is an alternative for the preservation of insecticidal compounds extracted from neem leaves.

## 1. Introduction

The increasing population worldwide demands the production of considerable amounts of food. Agricultural food production is a necessary activity to cover the feed demand. The trend in agricultural food production is to reduce the use of synthetic insecticides. Although these organic biocides are highly effective in controlling pests, they cause health problems to humans, contaminate soil and water, and are bioaccumulative [1]. 

The use of sustainable and environmentally friendly strategies safely protects the crops [2]. Therefore, research efforts have been focused on biocontrol, particularly through the use of plant extracts [3]. Worldwide, there is increasing interest in the use of botanical insecticides, evidenced by the increase in publicized papers. The production of biopesticide is led by China, USA, France, Brazil, and Japan, and the largest markets are Europe and Asia [4].

The neem (*Azadirachta indica* A. Juss, Meliaceae) is a plant originally from northeast India but is well adapted to regions such as America (Caribbean, central and south America), Asia (Nepal, Pakistan, Bangladesh, Sri Lanka, Myanmar, Thailand, Malaysia, Indonesia and Iran, China, Turkey, Indonesia), Africa (Cameroon), and even Queensland, Australia [5]. It is a versatile plant that has been reported as a source of insecticidal compounds such as nimbin, nimbidin, azadirachtin, salannin, thionemon, and meliantriol occurring in the seeds, leaves, and bark in high concentrations [6]. 

The components of the plant have shown insecticidal bioactivities at different levels when applied to crops and under laboratory conditions [7,8,9,10]. For example, the use of neem oil against *Bemisia tabaci* resulted in low mortality in both laboratory assays and field assays [11]. Li et al. [12] reported the use of neem leaf extracts against *Oxya chinensis*. The ethanolic extract showed a mortality of 26.67%. Furthermore, the insecticidal activity of neem was tested against *Leptinotarsa decemlineata*, and relatively high doses of neem extracts were required for the LC_50_ (75 mg/L) and LC_90_ (105 mg/L) [13]. The use of the cold-pressure extraction method and the liquid chromatography fractionation seems to be the method that enhances the insecticidal properties of neem [5].

However, most of the neem components are very sensitive to environmental factors, such as light, oxygen, and temperature, that cause degradation or volatilization [14]. Rapid degradation of the insecticidal compounds warrants injudicious or repetitive use at short intervals resulting in non-target impacts [15]. Hence, there is a need to develop strategies for preserving the biocontrol formulations obtained from plant extracts. 

The uses of encapsulation agents that wrap the components of the plant extracts are a current strategy for its preservation [16]. Many publications have demonstrated the efficiency of encapsulation processes in the preservation of neem compounds [17,18]. The encapsulation methods have involved the development of micrometric and nanometric particle sizes [14,19,20]. Complex coacervation is a method that allows microencapsulating water-immiscible liquids or water-insoluble solids by mixing two oppositely charged polymers [21]. Factors such as the pH and the ratio of polymers have been considered for successfully encapsulating bioactive compounds [22]. To encapsulate bioactive compounds, the encapsulating agents must be generally recognized as safe (GRAS) and biodegradable materials [20]. 

The encapsulation of bioactive compounds by the complex coacervation method is useful for the entrapment of hydrophobic compounds [23]. The presence of proteins and polysaccharides is necessary to form the complexes (capsules) [22]. Whey protein isolate (WPI) and pectin were used for the microencapsulation of neem leaf extracts in the present study. WPI is a mixture of globular proteins, including β-lactoglobulin and α-lactalbumin, with positively charged and uncharged amino acids; the protein chains are both hydrophobic and hydrophilic [24]. Polysaccharides, such as pectin, are negatively charged and facilitate protein interactions by electrostatic interactions [25]. However, the formation of stable complexes needs to be defined by the optimization of the encapsulation conditions, as do the protein/polysaccharide ratio and the pH required for the formation of complexes.

The Taguchi methodology is a tool reported for the optimization of different bioprocesses [26,27,28]. The methodology includes the use of orthogonal arrays that reduce the time and cost of the process by reducing the experimental runs. Furthermore, the Taguchi methodology improves the quality of the tested processes due to the versatile analyzing models [29]. 

The objective of the present work was to define the encapsulation conditions of *A. indica* extracts by analyzing their insecticidal activity. The coacervation method was used for entrapping the extracts into WPI–pectin microcapsules. The insecticidal activity of microcapsules was tested using *Tenebrio molitor* larvae as a model insect.

## 2. Results

### 2.1. Experimental Design and Mortality of T. molitor

The experimental design with the L9 array allowed us to evaluate the influence of the factors on the encapsulation process. Table 1 shows the results obtained experimentally following the Taguchi methodology. Run 3 reached the highest insect mortality (53.33%) and the highest S/N ratio (12.55). However, run 3 was not significantly different from treatments 2 (50% mortality and 12.43 S/N ratio) and 1 (36.67% mortality and 10.59 S/N ratio). The higher S/N ratio means high insect mortality and low variability of the experimentation. On the contrary, runs 7 and 8 did not affect the mortality of *T. molitor* after 48 h. It is important to note that the lower pH treatments had higher mortality and were reproducible (low variability). The controls used (polymers mixture and distilled water) showed no insect mortality. 

### 2.2. Individual Factor Contribution

**Figure 1:**
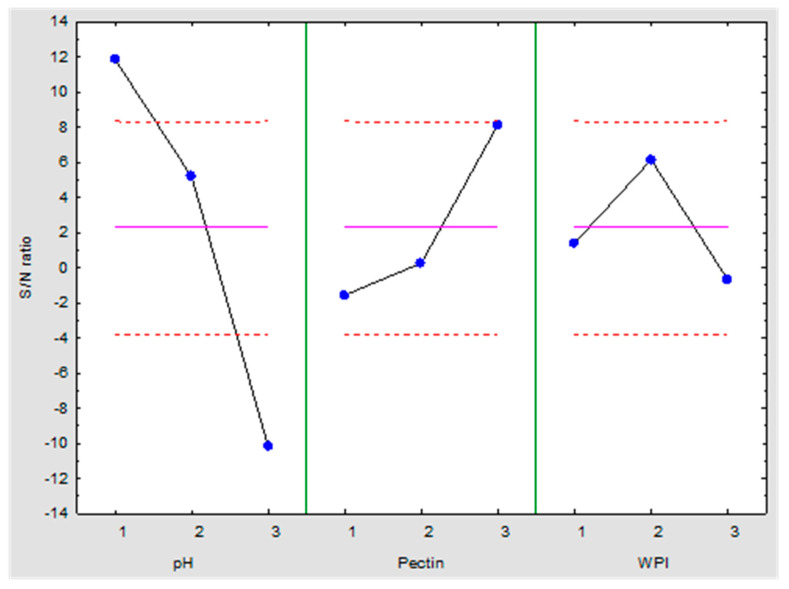
Individual factor performance at different levels. Dashed lines indicate ± 2 standard error, and S/N indicates signal-to-noise ratio.

The results obtained experimentally from the Taguchi L9 orthogonal array were analyzed in order to know the effects of the factors. Figure 1 shows the performance based on the S/N ratio of the individual factors at different levels. The pH showed better performance at level 1 (pH 3), and pH 6 showed a good S/N ratio value. However, by increasing to pH 9, a negative effect was obtained. This information agrees with the previous discussion related to the modification of charges in the WPI by increasing the pH values. On the contrary, the highest values of pectin were required for the encapsulation of insecticidal compounds. The individual performance of the WPI indicates that level 2 (6 g/100 mL) had a better effect on insect mortality than levels 1 (4 g/100 mL) and 3 (8 g/100 mL).

The analysis of variance (Table 2) obtained in the present study indicated that the most important factor in the encapsulation process was the pH, with an influence of 72.65%. Then, the pectin was second in importance (15.12% of influence), and finally, was the WPI (6.98% of influence). 

It is important to remark that an error of 5.25% was observed (Table 2). The Taguchi methodology considers, in the error term, not only the experimental error but also the factors not considered in the experimentation and uncontrollable factors [26,28]. However, a low error was obtained in the experimentation, indicating the good adaptation of the experiments to external factors [27].

### 2.3. Optimization of the Encapsulation Process and Validation

After analyzing the experimental data, the optimal encapsulation conditions were predicted based on the S/N ratio. The response was based on the mortality of *T. molitor*. The results obtained experimentally were analyzed by the higher equation. The analysis revealed that the optimal encapsulation conditions of neem leaf extracts were pH 3, 1 g of pectin, and 6 g of WPI (Table 3). By these conditions, the software Statistica 7 predicted an S/N ratio of 21.57. Experimentally, the value was not reached since the predicted S/N value means that insect mortality was over 100%. This was because the software predicted a higher mortality value mathematically. The experimental validation showed an insect mortality of 85 ± 10.49%, which was calculated as an S/N ratio of 18.54. The experimental mortality value was higher than the S/N values obtained in the experimental matrix. The S/N ratio obtained under optimal conditions indicates that the experimental validation provides high-quality results since reproducible data was obtained. No insect mortality was obtained as an effect of the mixture of polymers and pH. 

### 2.4. Morphology Analysis of Spray-Dried Microcapsules

**Figure 2:**
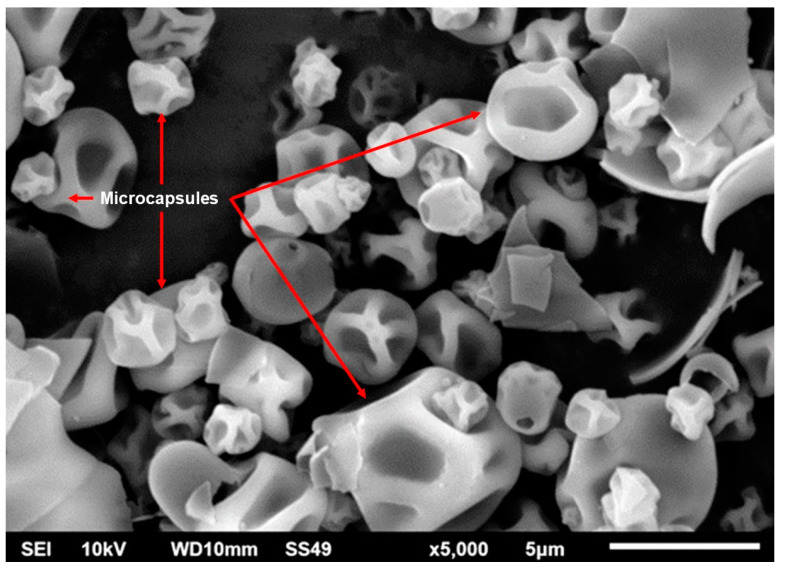
Scanning electron microscope image of the microcapsules. The image was viewed at ×5000, acceleration voltage of 10 kV, working distance of 10 mm, and scale of 5 μm.

The SEM analysis was carried out in order to know the morphology and size of the coacervates. Images obtained by SEM for the microcapsules are shown in Figure 2. The pectin–WPI complexes revealed irregular spheres of around 5 μm in diameter or smaller. The morphology was smooth on the surface but shrunk. The latter was an effect of the spray-drying process since the microcapsules were dehydrated. Furthermore, the type of wall material influences the morphology of the microcapsules. The size and shape of the microcapsules were not an issue for the main objective of the present work. The microcapsules are used to absorb the neem extract allowing the components to remain in the coating materials. 

## 3. Discussion

### 3.1. Neem Microcapsule Effect on the Insect Mortality

*A. indica* is a source of a wide range of compounds with diverse bioactivities. It includes the presence of nimbin, azadirachtin, flavonoids, terpenes, saponins, limonoids, alkaloids, tannins, and reducing sugars, among others [30]. The neem phytochemicals are extracted by using organic solvents such as ethanol, and the extracts have shown insecticidal activity [31]. Ethanol is a good option for the extraction of bioactive compounds since it is a no-harm solvent and is generally recognized as safe (GRAS) [29]. It is important to mention that in the present work, the organic solvent was fully recovered using a rotary evaporator. The solid-to-liquid ratio is also an important factor in the extraction process. This factor ranges from 1:10–1:16 *w*/*v* for the extraction of phytochemicals [27,29]. Thus, in the present study, we decided to use a solid-to-liquid ratio of 1:12 *w*/*v*. Most of the processes for the extraction of phytochemicals from *A. indica* are time-consuming, ranging from hours to days. The extraction of compounds, such as azadirachtin, by maceration can take 7 days [32]. However, in the present work, the extraction process was carried out for 24 h. The extraction conditions used allowed extracted insecticidal compounds from *A. indica*. 

The insecticidal activity varies according to the environmental conditions and the genetic variety of the neem due to the variation in the composition of phytochemicals [33]. Nevertheless, insecticidal activity is affected by environmental factors that decrease its bioactivity. The microencapsulation of bioinsecticides is an alternative to protect bioactive compounds. The microencapsulation of plant extracts with insecticidal activity has the advantage of reducing the impact on the environment and human health since the molecules used are biodegradable. Furthermore, the products can be directed to the target, the release of insecticidal compounds is controlled, and the bioactivity is maintained for a long period [18]. The neem extract microencapsulation has been effective for the biocontrol of insects. Costa et al. [17] evaluated the microencapsulation of neem using lignin obtained from sugarcane bagasse. The results evidenced that the microcapsules delayed the larva to the adult period and reduced the weight of the adults reducing the population of insects. Other works have been reported to achieve 100% mortality of *Spodoptera frugiperda* and *Diatraea saccharalis* by using microencapsulation [34]. This is a very important topic since the objective of biocontrol is to reduce the insect population to maintain environmental equilibrium. In the present work, during the development of the experimental design, it reached a maximum *T. molitor* mortality of 53.33% using microcapsules elaborated using WPI and pectin elaborated through the complex coacervation method. The treatments were tested against a model organism and under laboratory conditions. They provide information that helps to understand the effect of neem leaf extracts on similar insects. Although the mortality of *T. molitor* was monitored, the Taguchi S/N ratio was considered a response factor for statistical analysis. The S/N ratio assures the quality of the experimentation by analyzing the reproducibility. The higher the S/N value, the lower the variability [26,35]. 

### 3.2. The Effect of the Factors on the Microencapsulation Process

The complex coacervation method has been widely used for the preservation of plant bioactive compounds [36,37,38,39]. The method is based on the interaction of more than one polymer to form layers that protect the active compounds [40]. In the present work, the interaction of WPI and pectin were used for the formation of the complexes. The pH was the most important factor (73 % of influence) that affected the complex formation. As can be seen in Figure 1, it was possible to reduce the mortality of the insects by increasing the pH level. By increasing the pH value, the isoelectric point of the WPI was lost. This effect was caused due to the hydrogen groups (charged positively) being transformed into OH groups (charged negatively). Therefore, WPI is negatively charged. This generates repulsive forces with pectin, which is also charged negatively by the presence of carboxyl groups on the surface. In experiments with a higher level of pH, these repulsive forces were stronger than the electrostatic interaction between the polymers [41]. Hence, little insecticidal compounds were entrapped, causing low mortality of *T. molitor*.

On the contrary, by using the lower pH level, higher insect mortality was obtained. The explanation is that the amino groups of the WPI were protonated by decreasing the pH level below the isoelectric point (pI). This promoted the interaction with pectin (negatively charged) by electrostatic attraction [42]. The optimum pH level for the protein–pectin interaction depends on the constitution of the polymers [43]. 

The optimal conditions obtained from the statistical analysis (Table 4) were experimentally validated. According to the model employed in the statistical software, the expected S/N ratio was 21.57. Experimentally it was obtained that *T. molitor* mortality of 85 ± 10.49% corresponds to an S/N ratio of 18.54. Those results were 1.59-fold higher than those obtained in the experimental matrix, where the closest treatment obtained 53.33% mortality and an S/N ratio of 12.55. This confirms the high quality of the results due to the low variation. The S/N ratio is an important feature of the Taguchi methodology that distinguishes it from traditional experimental design methods. The S/N ratio can be used under three different conditions according to the Taguchi methodology, and they are ‘larger the better’, ‘smaller is better’, and ‘nominal is best’ [44]. The S/N values are estimated from the experimental outputs and are used to quantitively measure a response as a result of altering a parameter in the formulation process [45]. 

### 3.3. Morphology of Microcapsules

The morphology of the neem extract-loaded microcapsules was determined for the obtained microcapsules under the optimal conditions and is displayed in Figure 2. The particle sizes were a little variable (1–5 μm). The methodology used in the present work for the formation of the encapsulates was a modification of Ghasemi et al. [22]. They reported the nanoencapsulation of limonene. However, we could not reach sizes at the nanometric scale despite using a frequency of 40 kHz for 5 min. Perhaps the use of an ultrasonic probe instead of an ultrasonic bath can help to reach the nanometric scale. This is because the ultrasonic frequency is applied in close proximity to the treatments.

The WPI–carbohydrates complexes are used to vary shape when there are weak core-shell interactions [46]. In the present work, the shape was constant for all the microcapsules. The shape of the microcapsules was smooth on the surface but shrunk. The morphology, shape, and size of the microcapsules were expected to change after the spray-drying processes due to the conditions used. For the spray-drying instance, which utilized a temperature of 160 °C, the complexes showed irregular and shrunk morphologies due to the dehydration. It was because the microcapsule membrane is permeable to water vapor released by the spray-drying process [47], which causes the shrinking of the capsule. However, the extract was protected inside the coacervated microcapsules.

## 4. Materials and Methods

### 4.1. Plant Material and Insect Culture

The *A. indica* A. Juss (Meliaceae) leaves were obtained from the trees cultivated at Instituto Tecnológico de Ciudad Valles (22°1′15″ north, −99°2′16″ west). The leaves were harvested in the summer of the year 2021 (June–September). A total of 300 g of leaves were harvested in triplicate. The leaves were selected by size (8–10 cm), washed with abundant distilled water, dried at room temperature for 60 min, and used immediately.

*Tenebrio molitor* (Order, Coleoptera; Family, Tenebrionidae) larvae and wheat bran were purchased from the supplier tenebrios.com (Mexico City, Mexico). The larvae were placed into a plastic box (53.34 × 36.06 × 13.97 cm) containing a 2.5 cm bead of wheat bran as food. The insects were maintained at 28 ± 2 °C. For the assays, we selected 10–20 larval instars [48]. Every week we separated adults and pupae from the larvae and placed them in the conditions mentioned above to maintain the reproduction cycle.

### 4.2. Extraction Process

The *A. indica* leaves were size-reduced in a grinder (Mr. Coffee, Cleveland, OH, USA) to a particle size of 0.8–1.2 cm. The milled leaves were transferred to a flask containing aqueous ethanol (Jalmek®, San Nicolás de los Garza, Mexico) (50%) at a solid-to-liquid ratio of 1:12. The extraction was carried out by static maceration for 24 h, at room temperature (~25 °C), in dark, and in triplicate. The extract was filtered using filter paper Whatman No. 1 (GE Healthcare life sciences) and then microfiltered through PTFE 0.45 μm membrane (Whatman, Cytiva, Marlborough, MA, USA). The filtrate was concentrated in a rotavapor (Model R-200, Buchi, Flawil, Switzerland) by completely evaporating the ethanol. The total polyphenols concentration of the extract was quantified as reported by [29]. The gallic acid standard curve (0–100 μg/mL) was used as a reference. The results were expressed as gallic acid equivalents (GAE)/mL. The aqueous solution was used immediately for the encapsulation process.

### 4.3. WPI-Pectin Microcapsules Formation

WPI (Isopure®, Louisville, KY, USA) (isolate 80% protein) and pectin (JR foods) (low methoxy, esterification degree ≤ 50) were suspended individually in 50 mL of distilled water at the required concentration according to the experimental design. The WPI was stirred at 50 rpm for 15 min on a magnetic stirrer at room temperature. The pectin solution was heated at 50 °C and stirred at 150 rpm for 10 min and was tempered to room temperature. The solutions were stored at 4 °C overnight for complete solubilization. Then, both polymer solutions were mixed. Tween 80 was added at a ratio of 10% of the total solids into the solution and mixed to solve completely. The extract (100 mL, adjusted to 1 mg of GAE/mL) was added to the solution. The pH of the solution was adjusted as required (3, 6, and 9) using HCl and NaOH 0.1 and 1 N, respectively. Finally, the solution was homogenized using an ultrasonic bath (40 kHz) (Branson 3800) for 5 min.

### 4.4. Taguchi Methodology

The encapsulation process was carried out through the coacervation method. The Taguchi methodology was used for the optimization of the encapsulation process. The Taguchi method involves the establishment of a large number of experimental situations, described as an orthogonal array, to reduce experimental errors and enhance the efficiency and reproducibility of laboratory experiments [28]. The factors were considered as reported by Ghasemi et al. [22]. Tests were carried out on the impact of three main factors, each of which operated at three levels (Table 4).

The three factors and three levels allowed us to obtain the orthogonal array L9 (3^3^)—nine experimental runs in a combination of three factors at three levels. The experimentation was carried out as indicated in Table 2. Once we obtained the microcapsules, we applied every treatment to a total of 50 larvae. All treatments were developed in triplicate. The mortality of *T. molitor* was evaluated after 48 h. The experimental data were analyzed by the software Statistica 7 (Statsoft, Tulsa, OK, USA) through ‘the higher the better’ function. We considered the signal-to-noise (*S*/*N*) ratio as an indicator of the reproducibility and quality of the treatments according to the following equation:

$$S/N=-10\;Log10\;(\frac{1}{N}\times \sum \left(\frac{1}{y^{2}}\right))$$

where the factor −10 ensures that the ratio measures the inverse of “bad quality”, *y* represents the experimental value obtained in each trial, and *N* is the number of samples.

The analysis of variance (ANOVA) was carried out to obtain the contribution percentage of each factor that was determined as follows:

$$P=\frac{SSi}{SST}*100\;\%=\frac{SSi-MSi*dfi}{SST}*100\;\%$$

where *P* is the contribution percentage, *SSi* is the individual sum of squares, *SST* is the total sum of squares, *MSi* is the individual mean square, and *dfi* is the individual degrees of freedom value. 

The optimal encapsulation condition treatment was validated by evaluating the mortality after 48 h. The treatment was applied to a total of 50 insects, as mentioned next. All treatments were carried out in triplicate.

### 4.5. Insect Mortality Assay

The mortality of *T. molitor* was evaluated as a response factor in the present work, and the assays were carried out as reported by Loera-Corral et al. [49] with modifications. Once the extracts and microencapsulates were obtained, 30 mL was placed into a 100 mL beaker. Then, the larvae (instars 10–20) were immersed individually for 10 s to interact by contact with the coacervates. The larvae were allowed to dry for 5 min at room condition and were deposited into Petri dishes containing 3 g of wheat bran. The Petri dishes containing the larvae were stored at 28 °C. The mortality of the larvae was monitored after 48 h of the treatment application and was registered as the percentage of mortality of larvae. Water and the mixture of polymers were used as controls. The insect mortality data were analyzed by a Tukey test using the software Statistica 7 (Statsoft, OK, USA).

### 4.6. Morphological Characterization of the Microcapsules

The optimal treatment was submitted to scanning electron microscopy (SEM) analysis. Previously, the treatment was dehydrated and pulverized using a Spray-dryer (Buchi, Mini spray-dryer B-290, Switzerland). The equipment was adjusted to the following conditions: feed flow at 6 mL/min, the inlet temperature of 160 °C, and drying airflow at 40 m^3^/h. The powder was recovered and stored in hermetically sealed bags at room temperature until SEM analysis.

The morphological characteristics of the microcapsules containing the *A. indica* extracts were identified by scanning electron microscopy (SEM) using a JEOL JSM-6610LV Microscope (JEOL Inc., Peabody, MA, USA) with an acceleration voltage of 10 kV. Before imaging, each sample was sputter-coated with gold into a JEOL JFC-1100 sputter for 3 min. All the samples were processed and visualized at room temperature (20 °C). Digital images were captured using Quartz PCI imaging software v8 (Quartz Imaging Corp., Vancouver, British Columbia, Canada).

## 5. Conclusions

The Taguchi methodology is a useful tool that allowed us to find the optimal conditions for the microencapsulation of *A. indica* extracts based on the mortality of *T. molitor* larvae. The optimal conditions were obtained by analyzing the experimental results through the ‘the larger the better’ equation and using the S/N ratio. The pH was the most important factor (73% of influence) in the encapsulation process. The optimal encapsulation conditions showed a *T. molitor* mortality of 85 ± 10.49%, which means an S/N ratio of 18.54. The prediction was not reached (21.57 S/N ratio); however, the mortality rate ensured a decrease in the insect population. The microencapsulates had sizes ranging from 1 to 5 μm, and the morphology was shrunk with a smooth surface. The microencapsulation by complex coacervation of neem leaf extracts is an alternative for the preservation of insecticidal compounds. Further studies are necessary to evaluate the effect of the drying method on the efficiency of the microcapsules. Furthermore, it is necessary to evaluate the encapsulates against insects that affect crops. 

## Figures and Tables

**Table 1:** Experimental matrix for the Taguchi orthogonal array L9 (3^3^) and experimental results of S/N ratio and mortality of *T. molitor*.

No.	pH	Pectin	WPI	S/N Ratio	Insect Mortality (%)
1	1 (3)	1 (0.50)	1 (4.00)	10.59	36.67 ± 11.54 ^ab^
2	1	2 (0.75)	2 (6.00)	12.43	50.00 ± 20.00 ^a^
3	1	3 (1.00)	3 (8.00)	12.55	53.33 ± 15.17 ^a^
4	2 (6)	2	1	3.01	16.67 ± 5.77 ^ab^
5	2	3	2	3.67	26.67 ± 20.82 ^ab^
6	2	1	3	8.87	36.67 ± 20.80 ^ab^
7	3 (9)	3	1	0.00	0.00 ± 0.00 ^b^
8	3	1	2	0.00	0.00 ± 0.00 ^b^
9	3	2	3	3.01	16.67 ± 5.77 ^ab^
Control	-	-	-	-	0.00 ± 0.00 ^b^

WPI = whey protein isolate; S/N = signal-to-noise ratio; pH values = 3, 6, and 9; pectin values (g/100 mL) = 0.50, 0.75, and 1.00; WPI values (g/100 mL) = 4.00, 6.00, and 8.00. Control = insects immersed in distilled water. The same letters show no significant differences (Tukey *p* ≤ 0.05).

**Table 2:** Analysis of variance (ANOVA) for the three factors tested.

Factor	SS	dof	MS	F	*p*	Influence (%)
pH	765.68	2	382.84	13.84	0.07	72.65
Pectin	159.40	2	79.70	2.88	0.26	15.12
WPI	73.59	2	36.79	1.33	0.43	6.98
Error	55.32	2	27.66			5.25
Total	1053.98	8				100.00

**Table 3:** Optimal conditions for the microencapsulation of neem extract.

Factor	Level	Value	Effect Size
pH	1	3.00	9.60
Pectin (g/100 mL)	3	1.00	5.86
WPI (g/100 mL)	2	6.00	3.86
Predicted S/N ratio	21.57		
Experimental S/N ratio	18.54		
Insect mortality (%)	85.00 ± 10.49		
Control	0.00 ± 0.00		

WPI = whey protein isolate; S/N = signal-to-noise ratio; control = insects immersed in the polymer mixture and pH adjusted at optimal conditions.

**Table 4:** Factors and levels considered for the microencapsulation process.

No.	Factor	Units	Level 1	Level 2	Level 3
**1**	pH	-	3	6	9
**2**	Pectin	g/100 mL	0.50	0.75	1.00
**3**	WPI	g/100 mL	4.00	6.00	8.00

## Data Availability

The datasets used in the current study are available from the corresponding author on reasonable request.
